# Stable Loading of TiO_2_ Catalysts on the Surface of Metal Substrate for Enhanced Photocatalytic Toluene Oxidation

**DOI:** 10.3390/molecules28176187

**Published:** 2023-08-22

**Authors:** Le Xu, Jiateng Chen, Pengcheng Zhao, Boxiong Shen, Zijian Zhou, Zhuozhi Wang

**Affiliations:** 1 School of Energy and Environmental Engineering, Hebei University of Technology, Tianjin 300401, China; 2 A State Key Laboratory of Coal Combustion, Huazhong University of Science and Technology, Wuhan 430074, China

**Keywords:** TiO_2_, honeycomb aluminum plates, surface etching, binder, mechanism exploration

## Abstract

To promote the practical application of TiO_2_ in photocatalytic toluene oxidation, the honeycomb aluminum plates were selected as the metal substrate for the loading of TiO_2_ powder. Surface-etching treatment was performed and titanium tetrachloride was selected as the binder to strengthen the loading stability. The loading stability and photocatalytic activity of the monolithic catalyst were further investigated, and the optimal surface treatment scheme (acid etching with 15.0 wt.% HNO_3_ solution for 15 min impregnation) was proposed. Therein, the optimal monolithic catalyst could achieve the loading efficiency of 42.4% and toluene degradation efficiencies of 76.2%. The mechanism for the stable loading of TiO_2_ was revealed by experiment and DFT calculation. The high surface roughness of metal substrate and the strong chemisorption between TiO_2_ and TiCl_4_ accounted for the high loading efficiency and photocatalytic activity. This work provides the pioneering exploration for the practical application of TiO_2_ catalysts loaded on the surface of metal substrate for VOCs removal, which is of significance for the large-scaled application of photocatalytic technology.

## 1. Introduction

Volatile organic compound (VOC) has been considered as a kind of serious air pollution due to the toxicity and huge emission, which may lead to the formation of ozone, haze and fine particulate matter [1]. The annual emission of VOCs from industry reached 15.72 Tg in 2019 in China [2] and it is urgent to develop the efficient methods for VOCs removal from the exhaust gas. Photocatalytic VOC’s oxidation has been recognized as a potential technology for the elimination of VOCs due to its eco-friendliness and energy conservation, which realizes the VOC’s degradation under mild conditions by utilizing solar energy [3].

Currently, the researchers always deem that the key point of photocatalysis oxidation technology is the development of highly efficient photocatalysts [4], and many materials have been studied and adopted for photocatalytic VOCs oxidation in the laboratory [5,6,7]. However, the reports about the practical application of photocatalysts for VOCs removal are quite scarce. TiO_2_, as a typical semiconductor material, has been considered as the most likely photocatalyst for practical VOCs removal, due to its considerable oxidation ability, high chemical stability, non-toxicity and low price [8]. In the laboratory studies, the VOCs degradation efficiency of the modified TiO_2_ could reach above 90% [9], but the consideration of TiO_2_ in practical application is still lacking. In the practical industrial applications, it is impossible to use TiO_2_ powder directly due to the existence of high-speed gas flow in the exhaust gas [10]. The fixing of TiO_2_ powder on the suitable support to form a monolithic catalyst is important for the large-scaled VOCs photodegradation in the practical equipment. The support materials for the loading of TiO_2_ in the current reports are always the micro-nanomaterials, such as activated carbon fiber [11], molecular sieve [12] and SiO_2_ [13]. Although these support materials effectively improve the VOCs degradation efficiency of TiO_2_, the final products are still the powder catalysts and unsuitable for the practical application [14,15]. The high flow rate of actual exhaust gas demands for the highly stable loading of TiO_2_ powder on the surface of supports and the cost should also be considered. Nevertheless, the research about the suitable support for the loading of TiO_2_ to form a monolithic catalyst has not been reported and the stable loading method is worth an in-depth study, which is important for the practical application of photocatalytic VOCs oxidation technology.

Honeycomb aluminum plate has the advantages of stable structure, low cost and satisfactory dispersion for external forces, which is suitable as the support for the loading of TiO_2_ powder in the practical application [16]. The main limitation of honeycomb aluminum plate for the loading of TiO_2_ powder is the smooth surface, which leads to the easy dislodgement of TiO_2_ powder after the direct adhesion on the surface of plates. The recent studies indicated that improving the mechanical property of the supports can strengthen the interaction between catalysts and supports, and some surface treatment technologies have been proposed to improve the mechanical property of metallic materials, such as cold rolling [17], mechanical rolling [18] and shot peening [19]. Herein, the etching treatment was used to improve the surface roughness of aluminum plates for the efficient loading of TiO_2_ powder. In addition, the binder acts as a medium connecting the support and TiO_2_ powder, which is also a key factor affecting the loading stability. Common binders used for the loading of catalyst powder include water-based silicone resin, benzene propylene polymer, water glass and silica sol [20,21]. The organic solvents and water glass always lead to the cover of active sites [22], and silica sol always leads to the appearance of surface cracks [23], which further decrease the catalytic activity and life of catalysts. Hence, titanium tetrachloride was used as the binder in this work due to its highly dispersed homogenization and the strong chemical adhesion effect, which are beneficial to the stable loading of TiO_2_ powder on the surface of metal substrate.

In this work, different etching methods were used to change the surface property of honeycomb aluminum plate, and titanium tetrachloride was used as the binder for the loading of TiO_2_ powder. Toluene was selected as the typical representative of VOCs due to the widespread existence of monocyclic aromatic hydrocarbons in the actual exhaust gas. The loading stability of TiO_2_ powder and the photocatalytic activity of monolithic catalysts for toluene oxidation were further investigated. The optimal surface treatment scheme was proposed and the mechanism for the stable loading of TiO_2_ powder was revealed by experiment and DFT calculation. This work provides the pioneering exploration for the practical application of metal-substrate-loaded TiO_2_ for VOCs removal, which is of significance for the large-scaled application of photocatalytic technology.

## 2. Results and Discussion

### 2.1. The Loading Stability of TiO_2_ on Aluminum Plate

#### 2.1.1. The Loading Efficiency of TiO_2_ Powder

In order to investigate the effect of aluminum plate treatment method on the loading stability of TiO_2_ powder, the loading efficiencies of the monolithic catalysts were calculated. The mass of P25 (commercial TiO_2_ powder) used for the calculation of loading efficiency was 0.200 g. Table 1 shows the loading efficiency of TiO_2_ powder on the surface of aluminum plates treated by acid etching. Compared with the pristine aluminum plate, the acid etching with HNO_3_ solution could effectively improve the loading efficiency of TiO_2_ powder. When the impregnation time was selected as 5 min, the HNO_3_ concentration had a direct impact on the loading efficiency, which may be ascribed to the different surface roughness derived from acid etching. Therein, 15.0 wt.% HNO_3_ solution was the optimal condition to treat aluminum plate for the loading of TiO_2_ powder, which reached the highest loading efficiency of 36.6%. The excess HNO_3_ may lead to the destruction of surface structure, thereby decreasing the loading efficiency of TiO_2_ powder. Under the same HNO_3_ solution (15.0 wt.%), the effect of impregnation time on the loading efficiency was further explored. Therein, 15 min impregnation time was the optimal condition to treat the aluminum plate for the loading of TiO_2_ powder, which reached the highest loading efficiency of 42.4%. The excess impregnation time may also lead to the destruction of surface structure and then decrease the loading efficiency. Therefore, Ac-Al-15-15 exhibited the highest loading efficiency of TiO_2_ powder, indicating that the optimal surface treatment scheme by acid etching for the loading of TiO_2_ powder is the adoption of 15.0 wt.% HNO_3_ solution for 15 min impregnation.

Table 2 shows the loading efficiency of TiO_2_ powder on the surface of aluminum plates treated by anodizing etching. Compared with the pristine aluminum plate, the anodizing etching could also effectively improve the loading efficiency of TiO_2_ powder, which may be attributed to the improvement of surface morphology [24,25]. Under the same current (5 mA), the anodizing time had an impact on the loading efficiency of TiO_2_ powder, which may be ascribed to the different surface roughness derived from the formation of Al_2_O_3_. Therein, 15 min anodizing time was the optimal condition to treat aluminum plate for the loading of TiO_2_ powder, which reached the highest loading efficiency of 40.3%. The excess anodizing time may lead to the over-oxidation of aluminum plate and the destruction of structure [26], thereby decreasing the loading efficiency. Under the same anodizing time (15 min), the effect of current on the loading efficiency was further investigated. Therein, 5 mA current was the optimal condition to treat aluminum plate for the loading of TiO_2_ powder. Along with the increase in current, the loading efficiency integrally decreased and then kept relatively stable at the range of 10–30 mA due to the over-oxidation. When the current further increased, the loading efficiency sharply decreased due to the complete puncturing of aluminum plate [27]. Therefore, An-Al-5-15 exhibited the highest loading efficiency of TiO_2_ powder, indicating that the optimal surface treatment scheme by anodizing etching for the loading of TiO_2_ powder is the adoption of 5 mA current for 15 min anodizing. In addition, the loading efficiency of An-Al-5-15 was slightly lower than that of Ac-Al-15-15, indicating that acid etching has a slight advantage compared with anodizing etching.

#### 2.1.2. Analysis of Etching Rate

In order to prevent the irreversible destruction to the aluminum plates during the surface treatment process, the etching rates were calculated to analyze the loss of aluminum plates. The mass of aluminum plate was measured before and after the surface treatment process, and the etching rate was calculated as follows:

(1) $$\nu =\frac{∆W}{A\cdot t}$$

where ν is the etching rate, ∆W is the mass change before and after the surface treatment process, *A* is the contact area of aluminum plate (960 cm^2^) and *t* is the etching time.

**Figure 1:**
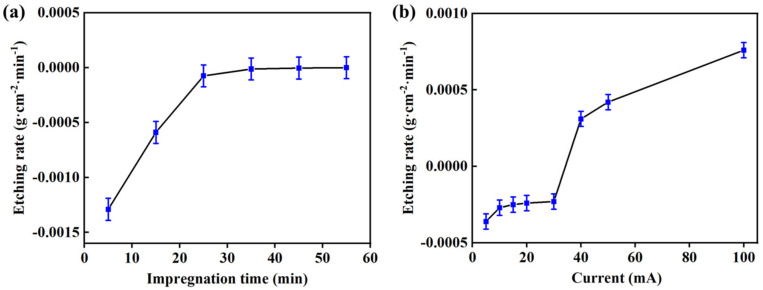
Etching rates of aluminum plates along with (**a**) impregnation time for acid etching and (**b**) current for anodizing etching (experimental conditions: acid etching with 15.0 wt.% HNO_3_ solution and anodizing etching for 15 min).

As shown in Figure 1a, the etching rate of aluminum plate treated by acid etching showed the increased tread before 25 min impregnation. After 25 min impregnation, the etching rate of aluminum plate remained unchanged around zero, indicating that the aluminum plate did not show the mass loss in the 15.0 wt.% HNO_3_ solution. As shown in Figure 1b, the etching rate of aluminum plate treated by anodizing etching basically remained unchanged when the current was lower than 30 mA after 15 min anodizing etching. When the current exceeded 30 mA, the etching rate of aluminum plate raised dramatically and showed the increased trend along with the increase in current, indicating that the aluminum plate showed the mass loss under the high current. Therefore, compared with anodizing etching, the acid etching in the suitable HNO_3_ solution could avoid the loss of aluminum plates, which is beneficial to be promoted in the practical application.

#### 2.1.3. The Shedding Efficiency of TiO_2_ Powder

**Figure 2:**
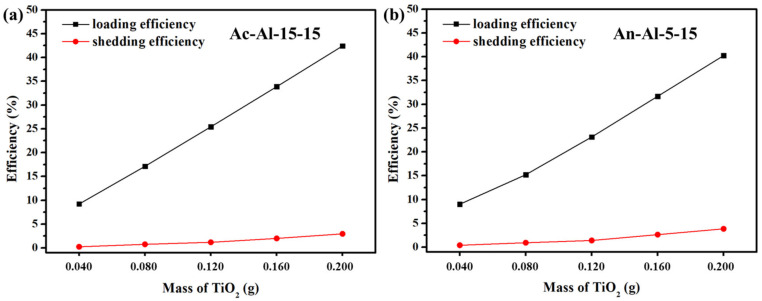
Loading and shedding efficiencies of TiO_2_ powder (0.040–0.200 g) on the surface of (**a**) Ac-Al-15-15 and (**b**) An-Al-5-15.

In order to investigate the loading stability of TiO_2_ powder on the surface of aluminum plates treated by different methods, a loading stability test was performed and the shedding efficiencies of the monolithic catalysts were calculated. Before the test, the aluminum plates were treated by acid etching in the 15.0 wt.% HNO_3_ solution for 15 min, or by anodizing etching under 5 mA current for 15 min. The dosage of P25 was also considered in the test, thereby 0.040, 0.080, 0.120, 0.160 and 0.200 g of P25 powder was used for the calculation of loading and shedding efficiencies. As shown in Figure 2, the loading and shedding efficiencies of TiO_2_ powder on the surface of Ac-Al-15-15 and An-Al-5-15 both increased along with the increase in P25 dosage, indicating that the loading and shedding amounts of TiO_2_ increased when the dosage of TiO_2_ increased. Meanwhile, the shedding efficiency was almost negligible compared with the loading efficiency, indicating that most of TiO_2_ powder could be immobilized on the surface of aluminum plate. When the loading efficiencies of TiO_2_ powder on the surface of Ac-Al-15-15 and An-Al-5-15 were 42.4% and 40.3%, the shedding efficiencies were only 3.0% and 3.9%. The shedding efficiency of TiO_2_ powder on the surface of Ac-Al-15-15 was slightly lower than that on the surface of An-Al-15-15, indicating that TiO_2_ powder could be immobilized more solidly on the surface of aluminum plate treated by acid etching. The results also indicated that the optimal surface treatment scheme of aluminum plates was the acid etched with 15.0 wt.% HNO_3_ for 15 min impregnation.

### 2.2. Surface Morphology of Aluminum Plate

**Figure 3:**
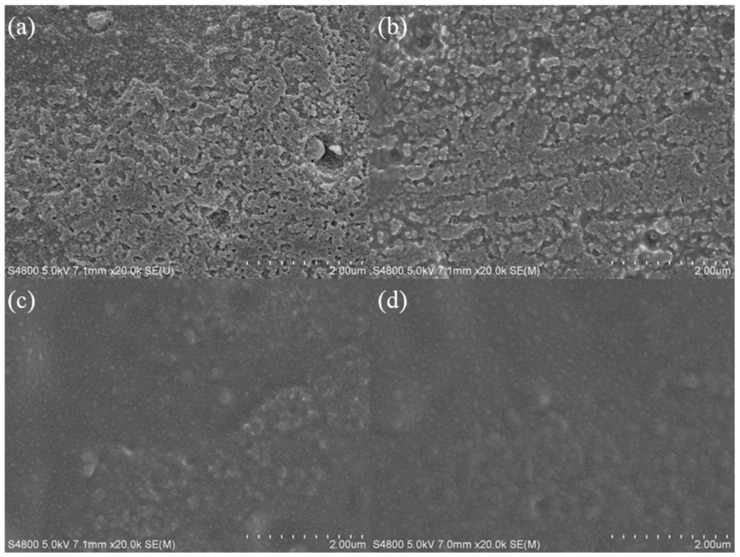
SEM images of the aluminum plates treated by acid etching: (**a**) Ac-Al-15-5, (**b**) Ac-Al-15-15, (**c**) Ac-Al-15-25 and (**d**) Ac-Al-15-35.

The surface morphologies of aluminum plates were observed by SEM to explore the effect of surface treatment method on the morphologies. SEM images of aluminum plates treated with 15.0 wt.% HNO_3_ solution are shown in Figure 3. As shown in Figure 3a, the aluminum plate showed an uneven and coarse surface after 5 min impregnation in HNO_3_ solution. After 15 min impregnation in HNO_3_ solution, the etching degree of aluminum plate in Figure 3b was intensified and the coarse surface remained. Meanwhile, the tiny pores increased compared with the pristine aluminum plate. Along with the further increase in impregnation time (25 min), the surface of aluminum plate in Figure 3c was over-etched and the surface structure was destroyed. The surface of aluminum plate became smooth due to the over-etching and it was not beneficial to the loading of TiO_2_ powder. After 35 min impregnation, the surface of aluminum plate in Figure 3d was completely destroyed, thereby leading to a smoother surface. Acid etching could obviously change the surface morphology of aluminum plates and the surface roughness increased after the suitable impregnation time. The results indicated that 15 min impregnation time is an optimal condition for the loading of TiO_2_ powder, which is ascribed to the coarse surface and verified by the loading stability test. The excess impregnation time could lead to the destruction of surface structure, which is not beneficial to the loading of TiO_2_ powder.

**Figure 4:**
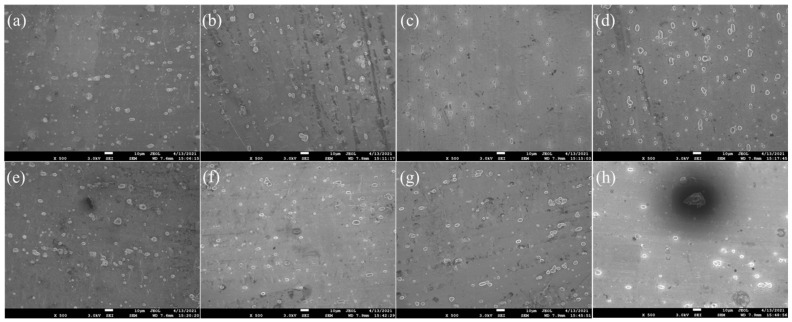
SEM images of the aluminum plates treated by anodizing etching: (**a**) An-Al-5-15, (**b**) An-Al-10-15, (**c**) An-Al-15-15, (**d**) An-Al-20-15, (**e**) An-Al-30-15, (**f**) An-Al-40-15, (**g**) An-Al-50-15 and (**h**) An-Al-100-15.

SEM images of aluminum plates treated by anodizing etching for 15 min are shown in Figure 4. As shown in Figure 4a,b, the aluminum plate showed the morphology with several dispersive particles on the surface, which is much different from the plate treated by acid etching, indicating that the anodic oxidation reaction successfully occurred on the surface of the aluminum plate. The formed particles on the surface could effectively increase the surface roughness, which is beneficial to the loading of TiO_2_ powder. As shown in Figure 4c,d, the pitting gradually appeared on the surface of aluminum plate when the current exceeded 10 mA, and the area and extent of pitting gradually expanded along with the increase in current. As shown in Figure 4e–g, the obvious pitting generated when the current exceeded 30 mA, indicating that the surface structure of aluminum plate was destroyed. As shown in Figure 4h, the aluminum plate had already been badly perforated when the current reached 100 mA. The results indicated that the small current could increase the surface roughness, but the excessive current could destroy the surface structure of aluminum plate. As a result, the aluminum plate treated by anodizing etching under 5 mA current showed the high loading efficiency of TiO_2_ powder and the high current led to a sharp decrease in loading efficiency.

**Figure 5:**
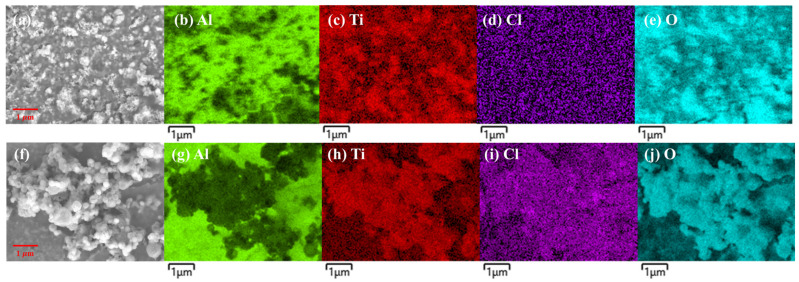
SEM image of (**a**) TiO_2_/Ac-Al-15-15 and the corresponding mapping images of (**b**) Al, (**c**) Ti, (**d**) Cl and (**e**) O; SEM image of (**f**) TiO_2_/An-Al-5-15 and the corresponding mapping images of (**g**) Al, (**h**) Ti, (**i**) Cl and (**j**) O.

To further investigate the loading of TiO_2_ powder, the surface morphologies of TiO_2_/Ac-Al-15-15 and TiO_2_/An-Al-5-15 were observed in Figure 5a,f. The element mapping images of Al, Ti, Cl and O for TiO_2_/Ac-Al-15-15 and TiO_2_/An-Al-5-15 are shown in Figure 5b–e and Figure 5g–j, respectively. The results indicated that TiO_2_ powder was successfully loaded on the surface of aluminum plates with the assistance of TiCl_4_. Meanwhile, TiO_2_ powder showed a uniform dispersion on the surface of Ac-Al-15-15, but an aggregation state on the surface of An-Al-5-15, indicating that the acid etching of plate is beneficial to the high dispersion of TiO_2_ powder, which accounted the high loading efficiency of TiO_2_ powder on the surface of plates treated by acid etching.

### 2.3. Photocatalytic Performance of the Monolithic Catalysts

**Figure 6:**
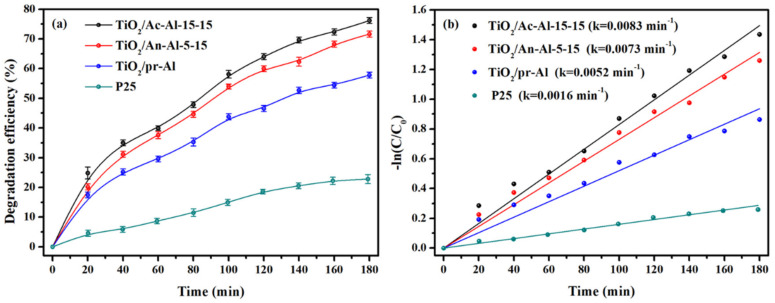
(**a**) Efficiency of photocatalytic toluene oxidation over different catalysts and (**b**) the fitting results by pseudo-first-order reaction kinetics.

The photocatalytic performance represents the actual possibility of the practical application for the monolithic catalysts. TiO_2_/Ac-Al-15-15, TiO_2_/An-Al-5-15 and TiO_2_/pr-Al were used for the photocatalytic activity test, and the dosage of TiO_2_ powder used for the activity test was 0.200 g. Meanwhile, single TiO_2_ powder (0.200 g) was also used in the photocatalytic activity test. Before the photocatalytic test, all catalysts were pretreated in dark to reach the toluene adsorption equilibrium. Then, the light source was turned on and the reaction was performed for 180 min. As shown in Figure 6a, the toluene degradation efficiencies of TiO_2_/Ac-Al-15-15, TiO_2_/An-Al-5-15, TiO_2_/pr-Al and P25 were 76.2%, 71.6%, 65.7% and 28.7% after 180 min reaction. The loading of TiO_2_ powder on the surface of aluminum plates could effectively strength the toluene degradation, which may be ascribed to the prevention of TiO_2_ powder aggregation. TiO_2_/Ac-Al-15-15 showed a higher toluene degradation efficiency than TiO_2_/An-Al-5-15 and TiO_2_/pr-Al, which was ascribed to the high loading efficiency of TiO_2_ powder. The kinetic constant (k) was further calculated by the pseudo-first-order model to reflect the reaction kinetics. As shown in Figure 6b, the k value of TiO_2_/Ac-Al-15-15 was 0.0083 min^−1^, which was about 5.0 times than that of single P25. Herein, TiO_2_/Ac-Al-15-15 showed the highest photocatalytic activity, indicating that the acid etching is an efficient method for treatment of aluminum plate to achieve the high loading efficiency of TiO_2_ powder, which facilitates the photocatalytic toluene oxidation.

### 2.4. Analysis of TiO_2_ Loading Mechanism

#### 2.4.1. Analysis of Substrate Surface

**Figure 7:**
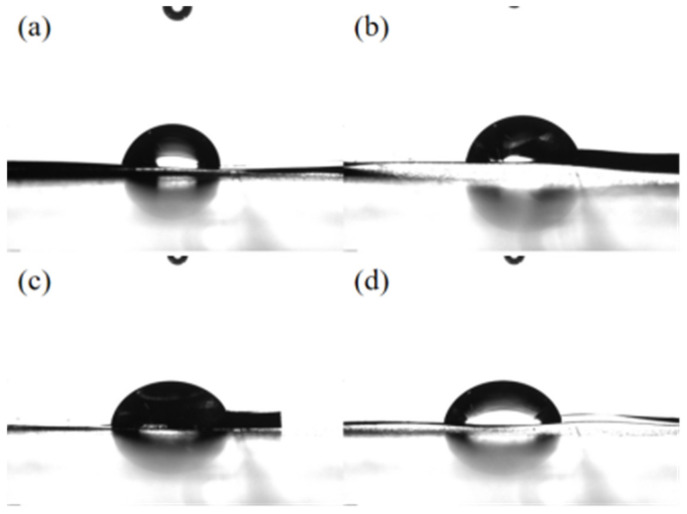
The contact angle of aluminum plates treated by acid etching: (**a**) Ac-Al-15-5, (**b**) Ac-Al-15-15, (**c**) Ac-Al-15-25 and (**d**) Ac-Al-15-35.

The surface roughness of an aluminum plate is an important factor for the stable loading of TiO_2_ powder. The surface contact angle and free energy were detected to clarify the effect of treatment methods on the surface roughness [28,29]. Deionized water was used as the titrate to measure the surface contact angle and free energy of aluminum plates treated by acid etching and anodizing etching. The contact angles of aluminum plates treated by acid etching for different impregnation time are shown in Figure 7a–d. Generally, the small contact angle indicates the strong interfacial interaction. The interface free energy increases along with the increase in contact angle, which reflects the deepening of surface roughness [30]. As shown in Table 3, the aluminum plate treated by 15 wt.% HNO_3_ solution for 15 min impregnation time showed the smallest contact angle and the highest free energy. As a result, Ac-Al-15-15 showed the high loading efficiency and photocatalytic activity.

**Figure 8:**
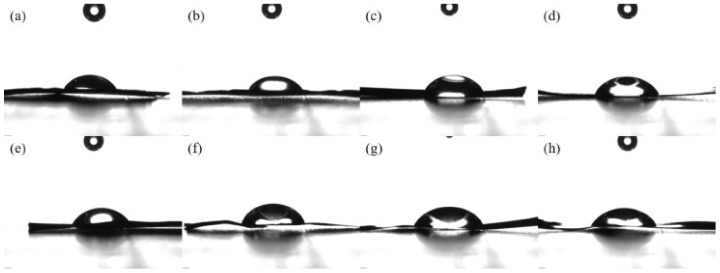
The contact angle of aluminum plates treated by anodizing etching: (**a**) An-Al-5-15, (**b**) An-Al-10-15, (**c**) An-Al-15-15, (**d**) An-Al-20-15, (**e**) An-Al-30-15, (**f**) An-Al-40-15, (**g**) An-Al-50-15 and (**h**) An-Al-100-15.

The contact angles of aluminum plates treated by anodizing etching with different current are shown in Figure 8a–h. As shown in Table 4, when the current was lower than 30 mA, the aluminum plates did not show the obvious surface pitting, and the aluminum plate treated by anodizing etching under 5 mA current for 15 min exhibited a relatively small contact angle and high free energy, indicating the increase in surface roughness and interaction between plate and binder. However, the contact angles of aluminum plates decreased and free energies increased when the current exceeded 30 mA. It was attributed to the appearance of obvious surface pitting under high current, which led to the destruction of surface structure in the SEM images. In spite of the small contact angle and the high free energy of aluminum plates under high current, the destruction of surface structure led to the low loading efficiency, which is not beneficial to the photocatalytic activity. Meanwhile, compared with the aluminum plates treated by anodizing etching, the smaller contact angle and higher free energy of plates treated by acid etching indicated the advantage of the acid etching method.

#### 2.4.2. The Interaction between Binder and TiO_2_

**Figure 9:**
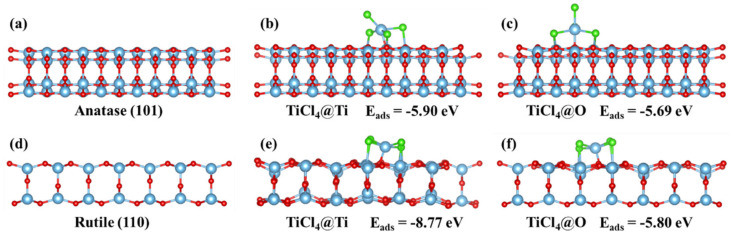
Optimized model of (**a**) anatase, (**b**) TiCl_4_@Ti site of anatase, (**c**) TiCl_4_@O site of anatase, (**d**) rutile, (**e**) TiCl_4_@Ti site of rutile and (**f**) TiCl_4_@O site of rutile.

The interaction between binder and TiO_2_ is also an important factor affecting the stable loading of TiO_2_ powder. The adsorption of TiCl_4_ molecule on the surface of TiO_2_ was explored by DFT calculation to clarify the interaction between binder and TiO_2_. The (101) crystal face of anatase and (110) crystal face of rutile were used for the construction of TiO_2_ surface model. As shown in Figure 9a,d, the (101) crystal face of anatase and (110) crystal face of rutile were selected as the highly active surface to perform the adsorption of TiCl_4_. Therein, Ti and O sites on the surface were considered as the active sites for the adsorption of TiCl_4_, and TiCl_4_ was placed above Ti and O sites for structure optimization. The optimized adsorption models on the surface of anatase and rutile are shown in Figure 9b,c,e,f. The adsorption energies of TiCl_4_ above Ti and O sites of anatase and rutile were −5.90, −5.69, −8.77 and −5.80 eV, respectively. Hence, the adsorption of TiCl_4_ on the surface of TiO_2_ could occur spontaneously and the surface of rutile could adsorb TiCl_4_ more easily. In addition, the TiCl_4_ molecule above the Ti site preferred to migrate and combine with adjacent O atoms. Due to the stable structure, the adsorption model in Figure 9b,e was selected for further calculation to reveal the loading mechanism.

**Figure 10:**
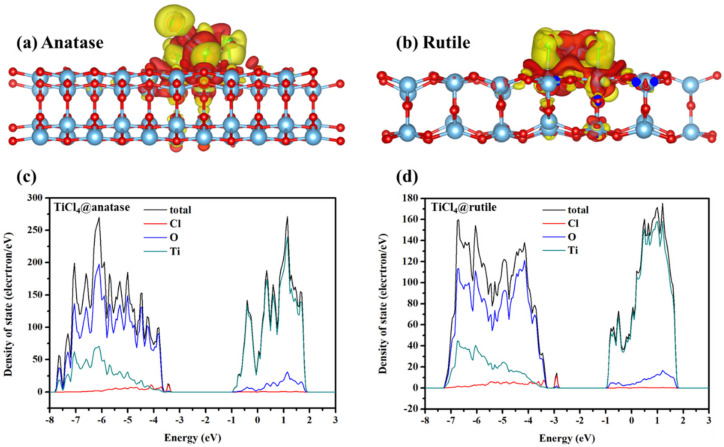
The differential charge density of TiCl_4_ adsorption model on the surface of (**a**) anatase and (**b**) rutile, where the isosurface level is set to be 0.0013 e/bohr^3^, and the regions of positive and negative charge are shown in yellow and red, respectively; DOS plots of TiCl_4_ adsorption model on the surface of (**c**) anatase and (**d**) rutile.

The differential charge densities of TiCl_4_ on the surface of anatase and rutile are shown in Figure 10a,b. The electrons preferred to distribute in the region closed to Ti atoms of TiCl_4_ or Ti atoms on the surface of TiO_2_, indicating the electron transfer from Cl atoms to Ti atoms. The results indicated that the chemical adsorption was established between TiO_2_ and TiCl_4_. The densities of state (DOS) for the adsorption model of TiCl_4_/TiO_2_ are provided in Figure 10c,d to further reveal the electronic structures. The results showed that the orbit of Cl atoms contributed the main intermediate band for the band structure, which led to the decreased band gap and indicated the existence of electron transfer between TiO_2_ and TiCl_4_. Mulliken charge distribution was further calculated to investigate the detailed electron transfer process. As shown in Table 5, the charge transfer direction was opposite for Ti and Cl atom, which is consistent with the above results. Therefore, the TiCl_4_ molecule is stable and chemically absorbed on the surface of TiO_2_ [31,32].

## 3. Materials and Methods

### 3.1. Treatment of Honeycomb Aluminum Plate

The industrial honeycomb aluminum plate was obtained from Galaxy Aluminum Co., Ltd. (Foshan, China). Before the pretreatment, the aluminum plate was tailored to the small cube with the size of 20 mm × 20 mm × 20 mm. The small aluminum plates were placed in the water bath at 100 ℃ for 6 min and then washed by deionized water. The final aluminum plates were obtained after drying overnight and used for the loading of TiO_2_. The mass of each aluminum cube was about 0.400 g. Two etching methods were used for the surface treatment of honeycomb aluminum plates:

Acid etching: Different concentrations of nitric acid solution (5.00, 15.0 and 25.0 wt.%) were prepared and the aluminum plates were placed in the solution for acid etching. The impregnation time was set as 5, 15, 25 and 35 min, respectively. After the impregnation, the plates were thoroughly washed with deionized water and dried at 80 °C for 10 h. The obtained plates were named Ac-Al-x-y, where x represents the concentration of HNO_3_ solution and y represents the impregnation time, respectively.

Anodizing etching: A three-electrode system provided the anodic oxidation environment for aluminum plates and nitric acid solution (15.0 wt.%) was used as the electrolyte. The current was set as 5, 10, 15, 20, 30, 40, 50 and 100 mA, respectively. The aluminum plates were treated for 5, 15, 25 and 35 min, respectively. After the anodizing, the plates were thoroughly washed with deionized water and dried at 80 °C for 10 h. The obtained plates were named as An-Al-x-y, where x represents the current and y represents the anodizing time, respectively.

### 3.2. The Loading of TiO_2_ Powder

Commercial TiO_2_ powder (P25) was selected as the active component to be supported on the surface of aluminum plates. Commercial titanium tetrachloride was selected as the binder for the loading of TiO_2_ powder. TiCl_4_ solution (0.1 mol/L) was purchased from Aladdin Biochemical Technology Co., Ltd. (Shanghai, China). The solution contained a small amount of hydrochloric acid to prevent the hydrolysis reaction. The aluminum plates were impregnated fully into the TiCl_4_ solution for several minutes. After that, the wet plate was taken out and P25 powder was sprayed to complete the loading of TiO_2_. The plates loaded with P25 powder were pre-cured at room temperature for 24 h and then dried at 100 °C for 2 h. The final plates loaded with TiO_2_ powder were named as TiO_2_/Ac-Al-x-y or TiO_2_/An-Al-x-y, where x and y represent the treatment conditions of aluminum plates. The pristine aluminum plate without etching was also used for the loading of TiO_2_ powder, which was named as TiO_2_/pr-Al.

### 3.3. Materials Characterization and Loading Stability Test

The morphologies of aluminum plates were observed by a scanning electron microscope (JSM-5900LV, Kyoto, Japan). The surface roughness of aluminum plates was tested by an optical contact angle tester (OCA200, Stuttgart, Germany). The loading stability test was performed to investigate the stability of TiO_2_ powder on the surface of aluminum plates. The plates loaded with TiO_2_ powder were placed in a drying beaker and then vibrated by an ultrasonic vibrator at 100 kHz for 60 min. After the thermal stabilization, the mass of monolithic catalyst was measured. The mass change of monolithic catalysts reflected the direct loading efficiency after the spray and the shedding efficiency after the vibration, which were calculated as follows:

(2) $$\eta_{l}=\frac{M_{1}-M_{0}}{M_{0}}\times 100\%$$

(3) $$\eta_{b}=\frac{M_{i}-M_{j}}{M_{i}}\times 100\%$$

where $\eta_{l}$ and $\eta_{b}$ represent the loading efficiency and shedding efficiency, $M_{0}$ is the mass of aluminum plate after the impregnation with titanium tetrachloride, $M_{1}$ is the mass of aluminum plate after the spray, $M_{i}$ is the mass of aluminum plate before the vibration and $M_{j}$ is the mass of aluminum plate after the vibration.

### 3.4. Photocatalytic Performance for Toluene Oxidation

Photocatalytic performance for toluene oxidation was tested in an off-line reactor (500 mL) at room temperature. The monolithic catalyst was placed on the bottom of the reactor and the mixed gas consisted of toluene, O_2_ and N_2_, which was injected into the reactor. The flow rates of N_2_ and O_2_ were 20 mL·min^−1^, while the flow rate of toluene with N_2_ as balance gas was 60 mL·min^−1^. The initial toluene concentration was stable at 60 ppm. After the adsorption in the dark for 1 h, the toluene concentration in the reactor was unchanged, indicating the toluene adsorption equilibrium, and then the reactor was sealed. The light source was provided by a 300 W Xe lamp (CEL-HXF300) with AM 1.5 G filter. Then, 1 mL of gas was sampled at 20 min intervals continually and a gas chromatography (2010 Plus, Shimadzu Corporation, Kyoto, Japan) was used for the measurement of toluene concentration. The photocatalytic degradation efficiency (“*η*”) was calculated as follows:

(4) $$\eta =\frac{C_{0}-C}{C_{0}}\times 100\%$$

where η is the degradation efficiency, $C_{0}$ is the initial concentration of toluene and C is the real-time concentration of toluene.

### 3.5. Computational Detail

Density functional theory (DFT) calculation was performed based on Device Studio PAW (DS-PAW) to explore the mechanism for the stable loading of TiO_2_ powder. Projector augmented wave (PAW) pseudopotential and generalized gradient approximation (GGA) in the form of Perdew–Burke–Ernzerhof (PBE) for exchange–correlation potentials were selected in the work [33,34]. The (101) crystal face of anatase and (110) crystal face of rutile were used for the construction of TiO_2_ surface model. A k-mesh of 1 × 2 × 1 was used for structure optimization and electronic property calculation. The convergence criterion for structural optimization was set to be 0.05 eV/Å. The adsorption energy (E_ads_) of TiCl_4_ was calculated by the following equation [35]:

E_ads_ = E_sub+TC_ − E_sub_ − E_TC_ (5)

where E_sub+TC_ is the energy of the optimized model, and E_sub_ and E_TC_ are the energies of TiO_2_ surface and TiCl_4_, respectively.

## 4. Conclusions

To promote the practical application of TiO_2_ photocatalysts for toluene oxidation, the honeycomb aluminum plates were selected the metal substrate for the loading of TiO_2_ powder. To strengthen the loading stability, surface etching treatment was performed and titanium tetrachloride was selected as the binder. The optimal surface treatment scheme was proposed and the mechanism for the stable loading of TiO_2_ was revealed by experiment and DFT calculation. The main conclusions are as follows:

(1) Acid etching of metal substrate has the advantage of high loading stability and photocatalytic activity compared with anodizing etching;
(2) The optimal surface treatment scheme of metal substrate is the acid etching with 15 wt.% HNO_3_ solution for 15 min impregnation;
(3) The high surface roughness of metal substrate and the strong chemisorption between TiO_2_ and TiCl_4_ account for the high loading efficiency and photocatalytic activity.

## Figures and Tables

**Table 1:** The loading efficiency of TiO_2_ powder on the surface of aluminum plates treated by acid etching.

HNO_3_ Concentration(wt%)	Impregnation Time (min)	Loading Efficiency (%)
none	none	20.8
5.00	5	30.5
15.0	5	36.6
25.0	5	33.5
15.0	15	42.4
15.0	25	39.2
15.0	35	38.0

**Table 2:** The loading efficiency of TiO_2_ powder on the surface of aluminum plates treated by anodizing etching.

Current (mA)	Anodizing Time (min)	Loading Efficiency (%)
none	none	20.8
5	5	35.2
5	15	40.3
5	25	38.6
5	35	31.4
10	15	34.6
15	15	34.6
20	15	35.5
30	15	36.5
40	15	30.4
50	15	28.5
100	15	28.2

**Table 3:** Surface contact angle and free energy of the aluminum plates treated by acid etching.

Impregnation Time (min)	Contact Angle (°)	Free Energy (J∙m^−2^)
5	68.3	35.0
15	61.9	37.7
25	71.1	33.7
35	72.0	32.7

**Table 4:** Surface contact angle and free energy of the aluminum plates treated by anodizing etching.

Current (mA)	Contact Angle (°)	Free Energy (J∙m^−1^)
5	76.1	31.8
10	83.4	29.8
15	90.9	28.8
20	77.3	30.9
30	71.3	36.5
40	72.8	36.2
50	74.8	36.4
100	63.0	37.2

**Table 5:** Mulliken charge distribution of TiCl_4_ adsorbed on the surface of anatase (101) and rutile (110).

Items	Species	s	p	d	Total	Charge/eV
TiCl_4_ adsorbed on the surface of rutile(110)	Ti	2.16	6.26	2.44	10.87	1.13
	Cl1	1.96	5.33	0	7.28	−0.28
	Cl2	1.96	5.33	0	7.28	−0.28
	Cl3	1.95	5.36	0	7.31	−0.31
	Cl4	1.95	5.32	0	7.27	−0.27
TiCl_4_ adsorbed on the surface of anatase(101)	Ti	2.19	6.34	2.45	10.97	1.03
	Cl1	1.94	5.33	0	7.27	−0.27
	Cl2	1.95	5.29	0	7.24	−0.24
	Cl3	1.95	5.28	0	7.23	−0.23
	Cl4	1.95	5.29	0	7.24	−0.24

## Data Availability

Data will be made available on request.
